# A STUDY ON CHANGES IN RESIDENTIAL LIFESTYLE AND AWARENESS OF RESIDENTS IN A HOUSING COMPLEX REGENERATION PROJECT

**DOI:** 10.1093/geroni/igz038.942

**Published:** 2019-11-08

**Authors:** tomoya ashihara, Akiko Nishino, yonggeun Lee, toshio otsuki, Kazuhiro nishide, yuji matsuda

**Affiliations:** The University of Tokyo, Tokyo, Japan

## Abstract

In Japan, where the ratio of elderly citizens exceeds 27%, there are issues such as the aging of residents and an increase in the number of people living alone. The present study aims to identify the elements required for a community base, based on temporal changes in daily life activities of residents in Housing Complex T, where more than 40% of residents are elderly, and in the surrounding area. A questionnaire survey was conducted with residents of Housing Complex T and residents in the surrounding area in 2010 (856/4,940 responses) and in 2018 (1,194/5,049 responses). The survey results showed that half of the elderly people participated in group circle activities during the day, and there was no change between 2010 and 2018. More than half of the residents of the housing complex utilize the supermarket within the housing complex several times a week. However, only 20% of the residents in the surrounding area use the supermarket. Parks were being utilized by 29.9% of housing complex residents and 37.6% of the residents outside of the complex, while the library was being utilized by 37.1% of housing complex residents and 39.1% of the residents outside of the complex. Public facilities are being utilized. These results show that it is important to provide architectural functions with high public utility within the property of housing complexes; they should be well known by residents of the surrounding area in order to create a community.

